# Hypoxia-Induced miR-148a Downregulation Contributes to Poor Survival in Colorectal Cancer

**DOI:** 10.3389/fgene.2021.662468

**Published:** 2021-05-31

**Authors:** Stepan Nersisyan, Alexei Galatenko, Milena Chekova, Alexander Tonevitsky

**Affiliations:** ^1^Faculty of Biology and Biotechnology, HSE University, Moscow, Russia; ^2^Faculty of Mechanics and Mathematics, Lomonosov Moscow State University, Moscow, Russia

**Keywords:** hypoxia, cobalt chloride, oxyquinoline, colorectal cancer, miR-148a

## Abstract

Hypoxia is an extensively investigated condition due to its contribution to various pathophysiological processes including cancer progression and metastasis formation. MicroRNAs (miRNAs) are well-known post-transcriptional gene expression regulators. However, their contribution to molecular response to hypoxia is highly dependent on cell/tissue types and causes of hypoxia. One of the most important examples is colorectal cancer, where no consensus on hypoxia-regulated miRNAs has been reached so far. In this work, we applied integrated mRNA and small RNA sequencing, followed by bioinformatics analysis, to study the landscape of hypoxia-induced miRNA and mRNA expression alterations in human colorectal cancer cell lines (HT-29 and Caco-2). A hypoxic microenvironment was chemically modeled using two different treatments: cobalt(II) chloride and oxyquinoline. Only one miRNA, hsa-miR-210-3p, was upregulated in all experimental conditions, while there were nine differentially expressed miRNAs under both treatments within the same cell line. Further bioinformatics analysis revealed a complex hypoxia-induced regulatory network: hypoxic downregulation of hsa-miR-148a-3p led to the upregulation of its two target genes, ITGA5 and PRNP, which was shown to be a factor contributing to tumor progression and poor survival in colorectal cancer patients.

## Introduction

Hypoxia is involved in the pathogenesis of colorectal and other cancers and is mainly associated with tumor growth, anti-apoptosis, recurrence, and poor survival (Brahimi-Horn et al., 2007; Vaupel and Mayer, 2007; Lange et al., 2014; Nagaraju et al., 2015). Multiple hypoxia-induced mechanisms are related to the activity of miRNAs—short non-coding RNAs whose main functional activity consists in post-transcriptional gene silencing (Cai et al., 2009). Interactions between miRNAs and their target genes were shown to play crucial roles in cell–cell communications (Turchinovich et al., 2015) and the pathogenesis of multiple diseases including, but not limited to, different types of cancer (Visone and Croce, 2009; Maltseva et al., 2014; Shkurnikov et al., 2019) and viral infections (Skalsky and Cullen, 2010; Nersisyan et al., 2020a).

Currently, there is no consensus on the regulation of miRNA expression by hypoxia: the effects heavily depend on cell types and the reason for hypoxia (either naturally or chemically induced), and only one miRNA, hsa-miR-210-3p, was found to be overexpressed under hypoxic exposure in the majority of reports (Kulshreshtha et al., 2007; Bavelloni et al., 2017; Bhandari et al., 2019). Multiple studies revealed a link between hypoxia- and cancer-induced miRNA expression change patterns: a large fraction of cancer-associated miRNAs can also be affected by hypoxia (Kulshreshtha et al., 2007; Shen et al., 2013; Panigrahi et al., 2018).

In this work, we studied the changes in miRNA and mRNA expression landscape of colorectal cancer cell lines Caco-2 and HT-29 exposed to hypoxia. Hypoxia was modeled by two different but widely used chemical agents: cobalt(II) chloride (CoCl_2_) and oxyquinoline, which cause long-term stabilization of hypoxia-inducible factor 1 and 2 (HIF-1 and HIF-2) (Wu and Yotnda, 2011; Osipyants et al., 2017; Muñoz-Sánchez and Chánez-Cárdenas, 2019; Savyuk et al., 2020). Such an experimental setup allowed us to precisely identify hypoxia-regulated miRNAs as well as their target genes. The role of the discovered interactions in colon cancer was further studied in patients’ tumors using The Cancer Genome Atlas Colon Adenocarcinoma (TCGA-COAD) cohort (Muzny et al., 2012)^1^.

## Materials and Methods

### Cell Cultures and Treatments

HT-29 cells (ATCC, Manassas, VA, United States) were grown in McCoy’s 5A medium, supplemented with 10% fetal bovine serum, 2 mM glutamine, 1% non-essential amino acids, penicillin (100 U/ml), and streptomycin (100 mg/mL).

Caco-2 cells were obtained from the Russian Vertebrate Cell Culture Collection (Institute of Cytology, Russian Academy of Sciences, St. Petersburg, Russia). The cells were incubated under conditions for differentiation for 21 days in Eagle’s minimal essential medium with 20% fetal bovine serum, 2 mM glutamine, 0.1 mM non-essential amino acids, and 0.1% penicillin–streptomycin.

All cell culture reagents were obtained from Gibco, Waltham, MA, United States. Both cell lines were maintained in a humidified atmosphere at 37°C and 5% CO_2_, changing the medium every 3 days.

A fresh stock solution of 0.3 M cobalt chloride (CoCl_2_) was prepared in water and added to the medium to obtain the desired final concentration 300 μM for 24 h. The second treatment included a fresh portion containing oxyquinoline derivative 4896-3212 (ChemDiv Research Institute, Khimki, Russia) in dimethyl sulfoxide (DMSO; 10 mM). The final concentration of oxyquinoline in the medium was 5 μM (0.5 μl of the solution in DMSO per 1 ml medium). In the control, the cells were incubated in a medium with 0.5 μl DMSO per 1 ml medium (without CoCl_2_ or oxyquinoline). Three biological replicates were used both for the control and the treated cells.

### RNA Extraction

Cells were lysed with the QIAzol Lysis Reagent (Qiagen, Hilden, Germany) for subsequent extraction of RNA using the Qiagen miRNeasy Mini Kit (Qiagen, Hilden, Germany). Nanodrop (Thermo Fisher Scientific, Waltham, MA, United States) was used to assess quality (260/280) and quantity. Total RNA samples were also QC-checked using the Agilent High Sensitivity DNA Kit (Agilent Technologies, Santa Clara, CA, United States).

### Library Preparation and Sequencing

Libraries for mRNA sequencing were prepared from total RNA samples using Illumina Stranded mRNA Library Prep Kit (Illumina, San Diego, CA, United States). Each sample was sequenced on the Illumina NextSeq 550 to generate single-end 75-nucleotide reads.

Libraries for miRNA sequencing were prepared from total RNA samples using NEBNext Multiplex Small RNA Library Prep Kit for Illumina. Each sample was sequenced on the Illumina NextSeq 550 to generate single-end 50-nucleotide reads.

### RNA-Seq Data Processing

The quality of FASTQ files was assessed with FastQC v0.11.9 (Babraham Bioinformatics, Cambridge, United Kingdom); three miRNA-seq replicates (control Caco-2, CoCl_2_-treated HT-29, and oxyquinoline-treated HT-29) had not passed the quality control (i.e., two, instead of three, replicates were used for these conditions in the downstream analysis). The adapters were trimmed with cutadapt v2.10 (Martin, 2011). The trimmed mRNA-seq reads were mapped on the reference human genome (GENCODE GRCh38.p13) with STAR v2.7.5b (Dobin et al., 2013). GENCODE genome annotation (release 34) (Frankish et al., 2019) was used to generate the count matrix. The miRNA count matrix was generated by miRDeep2 v2.0.1.2 (Friedländer et al., 2012) with the use of bowtie v1.1.1 (Langmead et al., 2009) and miRBase v22.1 (Kozomara et al., 2019).

The sequencing library sizes were normalized with the trimmed mean of *M*-values (TMM) algorithm, available in edgeR v3.30.3 package (Robinson et al., 2009), with default filtering of background noise. The same package was used to generate TMM-normalized fragments per kilobase of transcript per million mapped reads (FPKM) and reads per million mapped reads (RPM) matrices for mRNA-seq and miRNA-seq data, respectively. The obtained values were *log*_*2*_ -transformed. For further processing, we selected only highly expressed entries by cutting off the lower 25% of genes and 50% of miRNAs according to their median FPKM/RPM values in each experimental condition (Toung et al., 2011; Zhang X. et al., 2019). The thresholding value was higher for miRNAs since miRNA expression distribution is significantly biased toward several molecules with very high expression levels (Nersisyan et al., 2020b).

### Differential Expression and Enrichment Analyses

Differential expression analysis was conducted using DESeq2 v1.28.1 (Love et al., 2014); false discovery rates (FDRs) were calculated by the Benjamini–Hochberg procedure. For mRNA-seq data we performed testing of fold changes being above 1.5 using apeglm available in DESeq2 (Zhu et al., 2019), default 0.005 threshold was set on s-values. The resulting genes were uploaded to DAVID v6.8 (Huang et al., 2009) for enrichment analysis. For miRNA-seq data, differences with FDRs below the 0.05 threshold were considered.

### Prediction of miRNA Target Genes

At the first step of miRNA target prediction, we obtained the list of miRNA–gene interactions from miRDB v6.0 (Chen and Wang, 2020). The predictions were filtered according to their target scores; threshold value was set to 80. Then, we selected negatively correlated miRNA–gene pairs from the TCGA-COAD cohort (Muzny et al., 2012, see text footnote 1). Specifically, raw matched miRNA/mRNA sequencing count matrices of tumor samples (n=426) were downloaded from GDC Data Portal^2^ and converted to FPKM/RPM tables with the aforementioned TMM normalization. Then, Spearman correlation was calculated for each miRNA and predicted the target mRNA; 0.05 threshold was set on p-values. Additionally, we filtered out pairs with correlation values higher than −0.3 since they may reflect interactions that are too weak, though possibly statistically significant (Mukaka, 2012; Nersisyan et al., 2020c).

### Survival Analysis

Survival analysis (logrank test and Kaplan--Meier estimation) was conducted with the Python lifelines module^3^. Thresholds for defining groups of high and low gene expression were defined using the first and the third quartile of the corresponding distribution.

## Results

### Cobalt Chloride and Oxyquinoline Treatments Lead to Diverse Differential Gene Expression Landscapes

**FIGURE 1:**
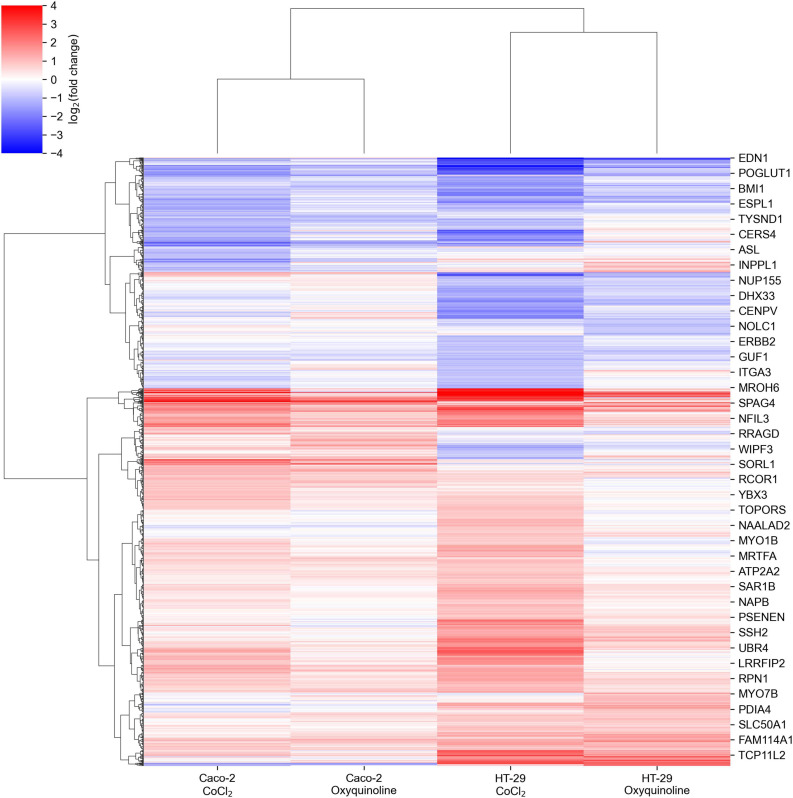
Changes in mRNA expression levels of colorectal cell lines exposed to chemically simulated hypoxia. A gene is included in the plot in case if it is differentially expressed in at least one out of four experimental conditions.

We performed mRNA sequencing of two colorectal cancer cell lines (Caco-2 and HT-29) exposed to CoCl_2_ and oxyquinoline treatments. In general, both treatments resulted in similar patterns of differentially expressed genes within each cell line, with a notably weaker effect of oxyquinoline (Figure 1 and Supplementary Table 1). Enrichment analysis of differentially expressed gene sets revealed a statistically significant activation of anaerobic glycolysis (KEGG pathway hsa00010 “glycolysis/gluconeogenesis”) and HIF-1 signaling pathway (KEGG pathway hsa04066 “HIF-1 signaling pathway”) in all experimental conditions, confirming the successive simulation of hypoxia. Interestingly, the level of HIF1A mRNA was increased in both treatments of HT-29 (2.6 folds for CoCl_2_ and 1.6 folds for oxyquinoline), while the increase was insignificant for Caco-2 (1.3 folds for CoCl_2_ and 1.06 folds for oxyquinoline). Such behavior was also reported in previous studies: while HIF-1 is mainly regulated *via* post-translational modifications, in some cell lines hypoxia may upregulate HIF1A at the transcriptional level (Catron et al., 2001; BelAiba et al., 2007).

**FIGURE 2:**
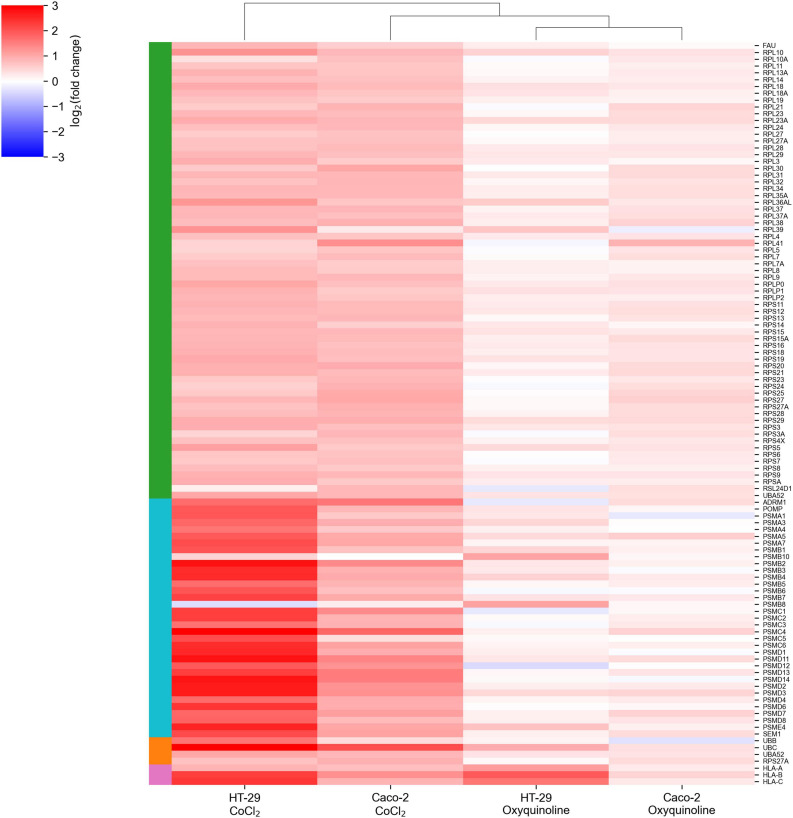
CoCl_2_-specific upregulation of gene groups. The green-, cyan-, orange-, and pink-colored rows are associated with ribosomal subunits, proteasome subunits, ubiquitin, and MHC class I molecules, respectively.

The major difference between treatments by two chemical agents consisted in a strong upregulation of genes encoding ribosomal proteins and genes involved in the major histocompatibility (MHC) class I antigen presentation pathway in response to CoCl_2_. This included almost all small and large ribosomal subunits, subunits of proteasome 26S, ubiquitin, and HLA-A, HLA-B, and HLA-C genes that make up MHC class I (Figure 2). As can be seen, these effects were negligible for oxyquinoline treatment. Thus, the cell can trigger an immune response upon CoCl_2_-induced oxidative stress *via* enhanced protein translation, proteasomal cleavage, and presentation of damaged peptides by MHC class I molecules. From the point of tumor hypoxia, this can be interpreted as activation of antitumor immunity.

### A Set of miRNAs Regulated by Chemically Induced Hypoxia Is Highly Dependent on Cell Line and Chemical Agent

**FIGURE 3:**
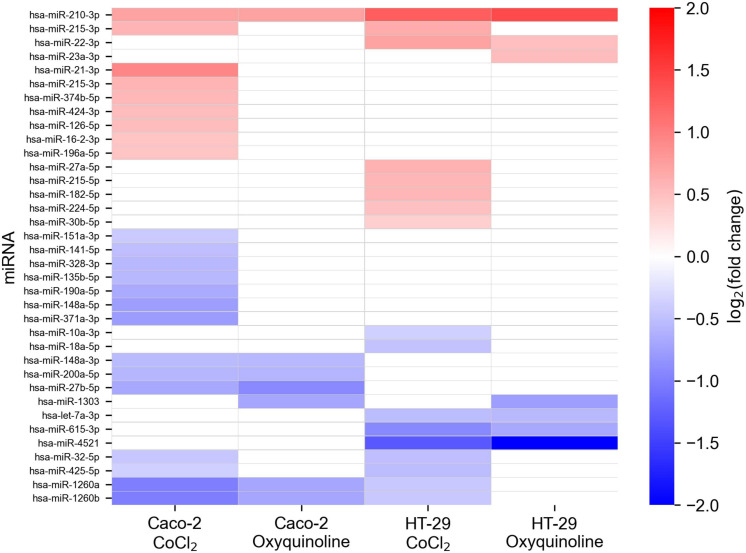
Changes in miRNA expression levels of colorectal cell lines exposed to chemically simulated hypoxia. A miRNA is included in the plot in case if it is differentially expressed in at least one out of four experimental conditions. Empty cells correspond to miRNAs which were not differentially expressed or had not passed the thresholds on expression level (i.e., less expressed).

In addition to mRNA sequencing, we performed miRNA-seq on the same cells, which allowed us to reconstruct the whole miRNA and mRNA expression landscape under chemically simulated hypoxia. For the analysis, we selected miRNAs with a high expression level and a significant rate of differential expression in at least one experimental condition. Generally, numbers of differentially expressed miRNAs in two cell lines and two treatments followed the same pattern as in the case of mRNA sequencing, indicating a stronger molecular response to CoCl_2_, namely, there were 22 and seven differentially expressed miRNAs for CoCl_2_ and oxyquinoline treatments of Caco-2, while treatments of HT-29 using the same agents resulted in 16 and 7 miRNAs, respectively (Figure 3 and Supplementary Table 2).

Only one miRNA was differentially expressed in all experimental conditions: hsa-miR-210-3p was consistently upregulated in both cell lines treated by both chemical agents. Nevertheless, multiple miRNAs were deregulated within the same cell line under different factors causing hypoxia: five miRNAs (hsa-miR-27b-5p, hsa-miR-148a-3p, hsa-miR-200a-5p, hsa-miR-1260a, and hsa-miR-1260b) were affected in Caco-2, and four miRNAs (hsa-let-7a-3p, hsa-miR-22-3p, hsa-miR-615-3p, and hsa-miR-4521) were identified in HT-29. Notably, these lists contained miRNA with an especially high level of expression: hsa-miR-148a-3p, which contributes to 6.9% of the whole Caco-2 miRNome, was 1.45-fold downregulated under both treatments of Caco-2. Treatment-specific miRNAs (i.e., miRNAs which are differentially expressed only under one treatment) were excluded from the downstream analysis.

### Downregulation of miR-148a Contributes to Poor Survival in Colorectal Cancer Patients by Upregulation of Its Target Genes

In order to identify targets of 10 differentially expressed miRNAs, we implemented a three-step procedure. First, we made a sequence-based miRNA target prediction using miRDB software. Then, we selected miRNA-induced interactions which can lead to degradation of target mRNA in colon adenocarcinoma samples. For that, we selected negatively correlated miRNA–target mRNA pairs using the set of 426 primary tumors derived from the TCGA-COAD project. Finally, the obtained list was intersected with the list of genes significantly upregulated or downregulated in the opposite direction to the respective miRNA fold change (in both treatments of the respective cell line).

**FIGURE 4:**
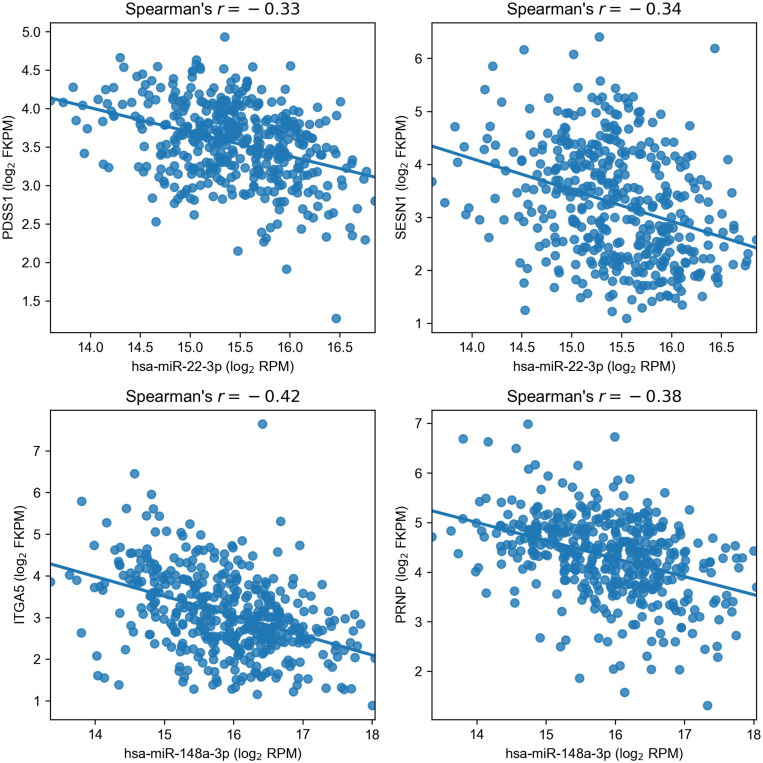
Negative correlation of miR-22 and miR-148a with their target genes in The Cancer Genome Atlas Colon Adenocarcinoma samples.

As a result, we obtained a list of four regulatory miRNA–mRNA interactions induced by two miRNAs: PDSS1 and SESN1 were targets of hsa-miR-22-3p (miR-22), and hsa-miR-148a-3p (miR-148a) regulated ITGA5 and PRNP (Figure 4). For additional confirmation on the existence of such interactions, we analyzed whether the target genes of miR-22 and miR-148a were deregulated only in the cell lines where the corresponding miRNA had altered the expression upon hypoxic exposure. The results fully supported our hypotheses: PDSS1 and SESN1 were not differentially expressed in Caco-2 (where miR-22 was also not differentially expressed), and PRNP was not differentially expressed in HT-29 (where miR-148a was not deregulated). Even stronger results were obtained for ITGA5: both treatments of Caco-2 induced the upregulation of three integrin alpha subunits (ITGA2, ITGA5, and ITGA6), and the expression increase for ITGA5 was much higher (18-folds for CoCl_2_ and 15-fold for oxyquinoline) compared to that of other subunits (3.44 folds at maximum). At the same time, ITGA5 expression was not changed in the HT-29 cell line, and ITGA2 was downregulated (i.e., the differential expression of integrins cannot be explained as a consequence of a regulation by one common factor).

**FIGURE 5:**
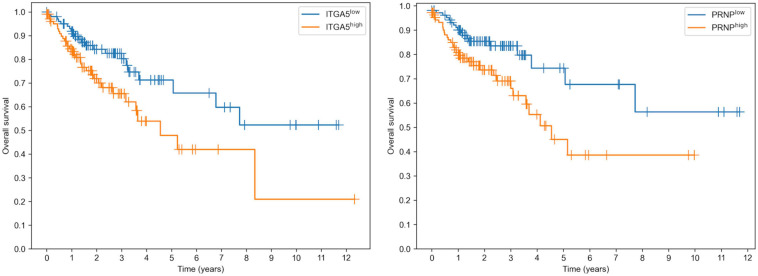
Prognostic value of ITGA5 and PRNP genes in The Cancer Genome Atlas Colon Adenocarcinoma cohort.

Surprisingly, both targets of miR-148a allowed us to predict the overall survival in colon adenocarcinoma patients with a statistical significance: logrank test p was equal to 0.0133 and 0.0119 for ITGA5 and PRNP, respectively (Figure 5). Moreover, overexpression of both genes led to poorer survival. In contrast, the expression levels of miR-22 targets (PDSS1 and SESN1) were not associated with overall survival. Thus, downregulation of miR-148a can lead to colon cancer progression through upregulation of its target genes.

### Hypoxia Could Induce the Downregulation of miR-148a Through TFAP2C Transcription Factor Activation

To assess whether the expression of miR-148a was decreased during hypoxia of patient tumors, we calculated Spearman correlation between the expression levels of miR-148a and two markers of hypoxia, HIF1A and SLC2A3 (Stuart Wood et al., 2007). This resulted in r=–0.22 (p=6.80× 10^−6^) for HIF1A and r=–0.3 (p=3.48× 10^−10^) for SLC2A3, supporting the hypothesis of hypoxia-induced downregulation of miR-148a.

**FIGURE 6:**
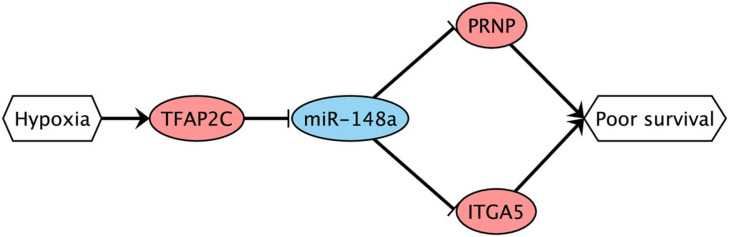
Possible hypoxia-driven regulatory network induced by miR-148a gene silencing. Red nodes are associated with upregulation; blue nodes are associated with downregulation. The arrows and T-shaped lines signify activation and repression, respectively.

Finally, we conducted bioinformatics analysis to predict possible transcription factors (TFs) which could directly regulate miR-148a during hypoxia. For that, we used the TransmiR database of TF–miRNA regulations supported by ChIP-seq experiments (Tong et al., 2019). Similar to what we have done with miRNA target prediction, we searched for TFs which are present in the database, deregulated under both treatments of Caco-2 in the same direction, and whose mRNA level significantly correlated with miR-148a in the TCGA-COAD samples (with the matching sign). As a result, we found transcription factor AP-2 gamma encoded by TFAP2C gene: its expression levels were increased by 10.2 and 6.6 times during CoCl_2_ and oxyquinoline treatments, and the correlation in TCGA-COAD was equal to −0.19 (p=2.02× 10^−3^). Meanwhile, this TF was not expressed in HT-29 cell lines. Taking all these together, we propose the possible complex hypoxia-induced regulatory network (Figure 6): hypoxia promotes the expression of TFAP2C transcription factor, which results in the decreased expression of miR-148a. The latter induces the upregulation of two miRNA target genes (ITGA5 and PRNP), which finally contributes to tumor progression.

## Discussion

Two different colorectal cell lines and two chemical agents were used to assess the effect of hypoxia on cellular mRNA and miRNA expression levels. As expected, hypoxia was induced in all four experimental conditions; however, there were both similarities and dissimilarities in the landscapes of differentially expressed mRNAs and miRNAs. In particular, CoCl_2_ induced a stronger deregulation on the transcriptomic level, promoting the expression of ribosomal subunits, proteasome subunits, ubiquitin, and MHC class I genes (Figure 2).

Consistently with previous reports, we observed the upregulation of miR-210 in both cell lines treated by both chemical agents (Kulshreshtha et al., 2007; Bavelloni et al., 2017; Bhandari et al., 2019). Aside from miR-210, we observed a differential expression of nine miRNAs within the same cell line under both exposures. One of those miRNAs, miR-148a, had a particularly high expression level contributing to approximately 7% of the whole miRNome of Caco-2. For both treatments, the expression levels of miR-148a were 1.45-folds decreased.

The multi-step bioinformatics analysis revealed two targets of miR-148a: integrin subunit α5 encoded by ITGA5 gene and the major prion protein PrP encoded by PRNP gene; both genes were upregulated in Caco-2 exposed to chemical hypoxia. Surprisingly, the elevated expression levels of both genes were associated with poor prognosis in colorectal cancer patients from the TCGA-COAD cohort. Thus, we hypothesize that direct interactions of miR-148a with ITGA5 and PRNP play an important role in tumor progression and metastasis. To the best of our knowledge, there are no studies on these interactions in colon cancer, though several groups already highlighted the role of miR-148a regulation of ITGA5 in other cancers, namely, such observations were made and experimentally verified (luciferase reporter assays) for breast (Cimino et al., 2013), gastric (Tseng et al., 2011), and non-small cell lung (Zhang J. et al., 2019) cancers. To the best of our knowledge, there are no reports containing a direct validation of interaction between miR-148a and PRNP. Aside from the mentioned target genes, it was already shown that miR-148a promotes apoptosis in colorectal cancer by silencing Bcl-2 (Zhang et al., 2011) and promotes proliferation of gastric cancer cells by targeting p27 (Guo et al., 2011).

In addition, we showed that the decreased expression of miR-148a is associated with tumor hypoxia in TCGA-COAD patients. With the use of TransmiR database of ChIP-seq experiments (Tong et al., 2019) and correlation analysis, we found a potential direct regulator of miR-148a during hypoxia: transcription factor AP-2 gamma encoded by TFAP2C gene. Further low-throughput experiments (such as overexpression/knockdown of miRNA/TF followed by reporter assay analyses) should be carried out to directly validate these hypotheses and study the proposed regulatory circuit in more detail. The proposed experiments should be also performed in more realistic *in vitro* microfluidic models (Samatov et al., 2015) and *in vivo*.

## Data Availability Statement

The datasets presented in this study can be found in online repositories. The names of the repository/repositories and accession number(s) can be found in the article/Supplementary Material.

## Author Contributions

SN, AG, and AT contributed to the conceptualization and methodology. SN, AG, and MC contributed to the formal analysis. SN and MC contributed to the writing—original draft. SN, AG, MC, and AT contributed to the writing—review and editing. All authors contributed to the article and approved the submitted version.

## Conflict of Interest

The authors declare that the research was conducted in the absence of any commercial or financial relationships that could be construed as a potential conflict of interest.

## Footnotes

1 https://portal.gdc.cancer.gov/TCGA-COAD

2 https://portal.gdc.cancer.gov/

3 https://zenodo.org/record/4457577

## Supplementary Material

Supplementary Table 1 The results of differential gene expression analysis.

Supplementary Table 2 The results of differential miRNA expression analysis.
